# The Signaling Pathway of PGE_2_ and Its Regulatory Role in T Cell Differentiation

**DOI:** 10.1155/2021/9087816

**Published:** 2021-11-26

**Authors:** Yang An, Jiameng Yao, Xiaoyin Niu

**Affiliations:** ^1^Department of Immunology and Microbiology, Shanghai Jiao Tong University School of Medicine, Shanghai Institute of Immunology, 280 South Chongqing Road, Shanghai 200025, China; ^2^Tongren Hospital, Shanghai Jiao Tong University School of Medicine, 1111 Xianxia Road, Shanghai 200336, China

## Abstract

Prostaglandin E2 (PGE_2_) is a lipid mediator derived from the fatty acid arachidonic acid. As an essential inflammatory factor, PGE_2_ has a critical impact on immune regulation through the prostanoid E (EP) receptor pathway. T cells, including CD4^+^ and CD8^+^ T cell subsets, play crucial roles in the adaptive immune response. Previous studies have shown that PGE_2_ is involved in regulating CD4^+^ T cell differentiation and inflammatory cytokine production via the EP receptor pathway, thereby affecting the development of diseases mediated by CD4^+^ T cells. In this review, we summarize the signaling pathway of PGE_2_ and describe the relationship between PGE_2_ and T cell differentiation. Hence, this review may provide important evidence for immune therapies and may even promote the development of biomedicines.

## 1. Introduction

Prostaglandin (PG) is a lipid mediator family derived from the fatty acid arachidonic acid (AA). Due to differences in their molecular structures, PGs were classified into nine groups, including PGA, PGB, PGD, PGE, PGF, PGG, PGH, PGI, and PGJ. Among these molecules, PGE_2_ is a kind of inflammatory factor that has been intensively studied. Although PGE_2_ has a short half-life and a 90% degradation rate in pulmonary circulation [1], it plays a significant role in mediating inflammation and multiple physiological processes [2]. PGE_2_ functions by prostanoid E (EP) receptors, which include four types of membrane-bound G protein-coupled receptors named EP1 to EP4 mediating the multiple signaling pathway. To date, a series of studies has demonstrated that PGE_2_ regulates the differentiation, maturation, and activation of immune cells, especially T cells [3, 4].

As essential components in the adaptive immune system, T cells are mainly divided into CD4^+^ helper T (Th) cells and CD8^+^ cytotoxic T lymphocytes (CTLs) [5]. After stimulation, CD4^+^ T cells can differentiate into a variety of effector subsets, including classical Th1 cells and Th2 cells, the subsequently defined Th17 cells, follicular helper T (Tfh) cells, and induced regulatory T (iTreg) cells. Th1 cells are characterized by their secretion of IFN-*γ* and are involved in cellular immunity, while Th2 cells produce IL-4, IL-5, IL-10, and IL-13 and are required for humoral immunity and allergic reactions. Th17 cells mainly secrete IL-17A, IL-17F, IL-21, and IL-22 and mediate inflammatory response. Tfh cells are a new subset of helper T cells that produce IL-21 and regulate the maturation of B cell responses. Treg cells are characterized by the expression of the forkhead transcription factor (Foxp3) and have essential roles in the maintenance of immune homeostasis by suppressing these effector T cell responses [6]. CTLs play their roles by directly killing infected cells and cancer cells. In this review, we provide a general overview of the metabolism of PGE_2_, the signaling pathways of EPs, and the regulatory role of PGE_2_ in T cell differentiation.

## 2. PGE_2_ Synthesis and Metabolism

**Figure 1:**
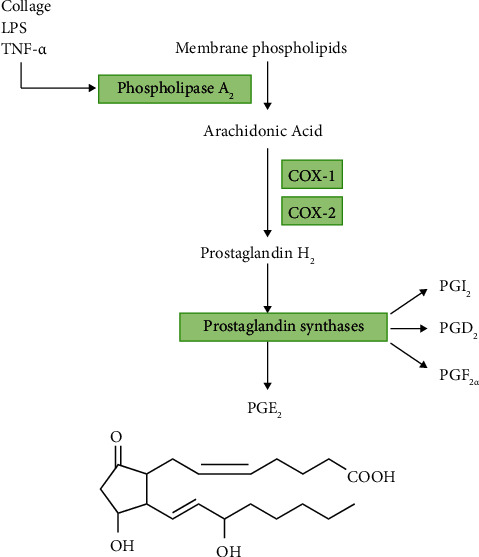
The process of PGE_2_ synthesis. Arachidonic acid is released from the membrane by phospholipase A_2_. Cyclooxygenases (COX-1 and COX-2) produce prostaglandin H_2_ (PGH_2_) from arachidonic acid. PGH_2_ is produced by prostaglandin synthase to produce PGI_2_, PGD_2_, PGE_2_, and PGF_2*α*_.

Prostaglandins are derived from AA and synthesized mainly through the cyclooxygenase (COX) pathway (Figure 1). When cells are damaged or stimulated, AA is released from plasma membrane phospholipids [7]. Free AA can be modified into prostaglandin or leukotrienes [8]. For prostaglandin synthesis, AA released from membranes is oxidized by the cyclooxygenase enzymes COX-1 and COX-2 [9, 10]. The enzymatic effect of COX enzymes on AA involves two steps, one is the oxidation of AA to form prostaglandin G2 (PGG_2_), and the other is the reduction of PGG_2_ to form prostaglandin H2 (PGH_2_). Depending on the specific enzymes, PGH_2_ can be modified to five different prostaglandins, including PGD_2_, PGI_2_, PGF_2*α*_, PGE_2_, and thromboxane A_2_ (TXA_2_) [2, 7]. PGE_2_ synthesis through COX is mediated by three distinct enzymes, cytosolic PGE synthase (cPGES), microsomal PGE synthase-1 (mPGES-1), and mPGES-2 [2].

Nearly all types of cells and tissues can secrete PGs. When PGs are secreted, however, they are quickly degraded by the lung or liver. The lung is the primary organ involved in PGE_2_ metabolism [11, 12]. PGE_2_ metabolism mainly occurs via three mechanisms, leading to the inactive metabolite: (i) 15-hydroxyprostaglandin dehydrogenase (15-PGDH) converts a 15-hydroxyl group to a keto group for metabolism; (ii) 9-ketoreductase reduces 9-ketone to hydroxyl; and (iii) a side chain is inactivated by *β*-oxidation and/or *ω*-hydroxylation. The final metabolites of PGE_2_ are eliminated from the body in the urine. The effects of PGE_2_ are regulated by the balance between its COX-2-regulated synthesis and 15-PGDH-driven degradation [13].

### 2.1. PGE_2_ Signaling and EP Receptors

After PGE_2_ is synthesized in the cytoplasm, it diffuses out of the cell through facilitated diffusion. Since PGE_2_ is quickly degraded, it is distinct from typical hormones that act on distant target tissues but can only be produced and released locally. After being secreted from cells, the lipid mediator binds to membrane receptors, activates downstream signaling pathways, and exerts biological effects [14].

**Figure 2:**
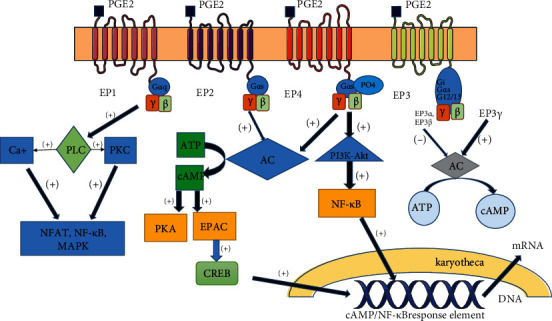
The signaling pathway of PGE_2_. The schematic diagram shows the downstream signaling pathway of PGE_2_. The lipid mediator actives or inhibits the target cell via four EP receptors, EP1, EP2, EP3, and EP4. Different receptors activate different signaling pathways which are connected in a network in the cell and cooperate with each other in vital processes.

PGE_2_ signals through four morphologically distinct G-protein-coupled receptors, namely, EP1, EP2, EP3, and EP4, and triggers divergent signaling pathways (Figure 2), thereby mediating its various biological functions. The mRNAs of the EP receptors also exhibit different expression patterns in a number of tissues, and activating each receptor subtype leads to distinct functional consequences [15]. EP3 and EP4 are high-affinity receptors and are expressed in all human tissues, whereas the activation of EP1 and EP2 occurs in only a few organs and requires significantly higher levels of PGE_2_ [16]. EP receptors are also expressed on various immune cell membranes. EP2 and EP4 are the main receptors of PGE_2_ involved in regulating the differentiation of CD4^+^ T cells [17]. By coupling with the activated G protein, it stimulates adenylate cyclase (AC) to activate the cAMP/protein kinase A (PKA)/cAMP-responsive element-binding protein (CREB) signaling pathway and downstream molecules to participate in mediating proinflammatory responses and inhibiting PGE_2_ activity [18]. It is well known that clear signal transduction of EPs is beneficial for therapeutic strategies against diseases related to the immune system.

### 2.2. EP1 Receptor

In humans, the EP1 receptor has the lowest affinity for PGE_2_ [19] and couples to a Gq alpha subunit (G*α*q). PGE_2_ can result in smooth muscle contraction through this complex. The human single EP1 receptor consists of 402 amino acid residues, whereas the rat single EP1 receptor consists of 405 amino acid residues. The EP1 receptor has 7 hydrophilic transmembrane domains. An arginine residue exists in the 7^th^ domain, and this residue is an important PGE binding site. The phosphorylation of PGE_2_ depends on cyclic adenosine monophosphate- (cAMP-) dependent protein kinase and protein kinase C (PKC). The G*α*q subunit activates phosphoinositide-phospholipase C (PLC) and finally leads to an elevation in intracellular Ca^2+^ and the activation of PKC, mediating gene transcription through the activation of the nuclear factor of activated T cells (NFAT), nuclear factor-kappaB (NF-*κ*B), and the mitogen-activated protein kinase (MAPK) pathways [16].

EP1 receptor mRNA is highly expressed in the kidney, lung, and stomach [15]. EP1 has not been demonstrated to play a significant role in innate immunity but participates in controlling T cell differentiation [2, 4].

### 2.3. EP2 Receptor

The human EP2 receptor consists of 358 amino acids [20]. This receptor is coupled to a Gs alpha subunit (G*α*s). The long C-terminal tail of the EP1 receptor contains serine and threonine, the phosphorylation of which depends on cAMP-dependent protein kinase [15, 21]. Gs proteins lead to an increase in cAMP and activation of PKA. A high concentration of intracellular cAMP can also activate both PKA and the exchange protein directly activated by cAMP (EPAC). Then, EPAC phosphorylates the transcription factor CREB [22]. In addition, EP2 receptor signaling leads to the inhibition of glycogen synthase kinase-3 (GSK-3), which inhibits the translocation of *β*-catenin into the nucleus [21].

The EP2 receptor participates in most of the immunoregulatory effects of PGE_2_ in both the innate and adaptive immune responses. For example, PGE_2_ in the supernatant of thyroid cancer cells inhibits the activity of NK cells through the EP2 and EP4 receptors [23]. In addition, PGE_2_ promotes neurite outgrowth and suppresses cell proliferation by activating the EP2 receptor subtype, and the cAMP signaling pathway is involved in the PGE_2_-induced differentiation of NSC-34 cells [24].

### 2.4. EP3 Receptor

The EP3 receptor, which consists of 365 amino acid residues, is an abundantly and widely expressed EP receptor [15]. EP3 also contains two cAMP-dependent protein kinase phosphorylation sites. EP3 is the only EP receptor that includes multiple variants (*α*, *β*, and *γ*). During the transcription of the EP3 coding sequence, several types of mRNA spliceosomes are produced. The differentiation of these spliceosomes results in a change in the C-terminal tail [25]. It should be noted that these variants have a similar affinity for PGE_2_, but they have different signal transduction pathways, relative expression patterns, and desensitization properties [2]. EP3*α* and EP3*β* are coupled to Gi and inhibit adenylyl cyclase (AC) or cAMP, whereas EP3*γ* is coupled to Gs or Gi and stimulates cAMP production. Therefore, a high concentration of PGE_2_ inhibits AC, while a low concentration of PGE_2_ activates AC [26].

EP3 mRNA can be detected in almost all tissues. EP3 signaling participates in many metabolic processes. The selective inhibition of EP3 might be a potential approach for reducing chronic neuropathic pain [27]. Moreover, it has been reported that EP3 signaling is induced in placentas associated with unexplained recurrent pregnancy losses [28].

### 2.5. EP4 Receptor

The EP4 receptor signals through a pathway similar to that of the EP2 receptor. The EP4 receptor consists of 513 amino acids. Similar to EP2, EP4 couples to the G*α*s protein and activates the cAMP/PKA pathway. cAMP is rapidly degraded by phosphodiesterase to limit the duration of the signal. PKA then phosphorylates target proteins in the cells [29]. EP2 exhibits higher sensitivity and can more effectively increase cAMP. However, the EP4 receptor has a higher affinity for PGE_2_ than the EP2 receptor [21]. Compared to EP2, EP4 performs the remarkable function of activating the phosphatidylinositol 3 kinase (PI3K) signaling pathways. The subsequent phosphorylation is mediated via G-coupled receptor kinases or via the ability to bind Gi protein [30, 31]. In addition, EP4 also stimulates noncanonical activation of the PI3K-Akt (also known as protein kinase B) and extracellular regulated kinase (ERK) pathways [16, 29].

Among all four types of EP receptors, the maintenance of EP4 has received much attention ^[32]^. PGE_2_-EP4 interaction causes muscle-specific stem cell expansion by triggering a cAMP/pCREB pathway that activates the proliferation-inducing transcription factor *Nurr1* [33]. Besides, activation of EP4 may mediate many cellular responses, such as the promotion of angiogenesis, proliferation, motility, and metastasis or the delay of tumor cell apoptosis [32].

### 2.6. Effects of PGE_2_ on T Cell Subsets

**Figure 3:**
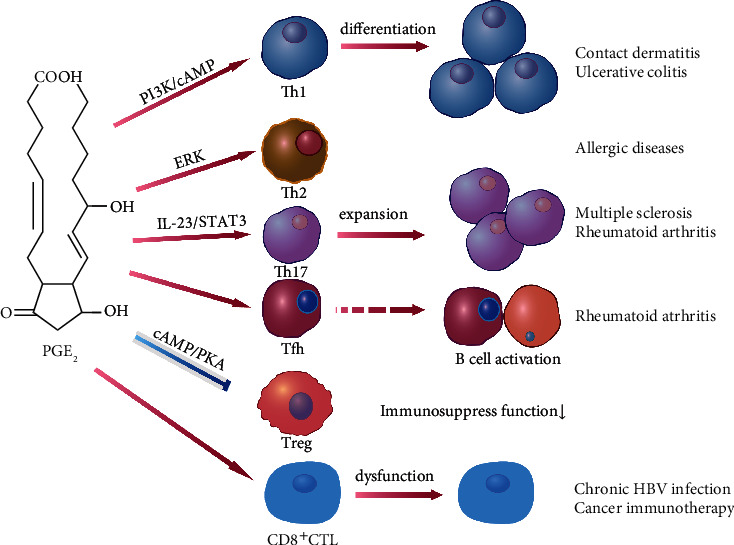
Regulation of T cell subsets by PGE_2_. PGE_2_ activates the differentiation of Th1 cells by decreasing the levels of cAMP; PGE_2_ can enhance the IL-4/IFN-*γ* ratio in CD4^+^ T cell culture and regulates CD4^+^ T cell polarization towards Th2 cells; PGE_2_ promotes the differentiation of naïve CD4^+^ T cells into Th17 cells via the EP2 or EP4 receptor and cAMP pathway; PGE_2_ may promote Tfh differentiation; PGE_2_ suppresses the EP/cAMP/PKA pathway and inhibits Treg cell differentiation; PGE_2_ inhibits the antiviral activity of CTLs and the survival of patients receiving cancer immunotherapy.

PGE_2_ exerts many complex immunoregulatory roles under physiological and pathophysiological conditions [34, 35]. PGE_2_ influences the differentiation of effector T cells, such as Th1, Th2, Th17, Treg, Tfh, and CTLs [3] (Figure 3).

### 2.7. PGE_2_ and Th1 Cells

Th1 cells mainly secrete IFN-*γ*, IL-2, and TNF-*α*, mediating cellular immune responses and playing a key regulatory role in resistance to bacterial and viral infections. T-bet is the key transcription factor of this T cell subset. Previous experiments indicated that both EP2- and EP4-mediated Th1 differentiation are inhibited by PI3K inhibitors [36]. This signaling pathway can decrease the cAMP level and then reduce the cAMP-mediated inhibition of T cell, indicating that PGE_2_ could activate the differentiation of Th1 cells. Other researches suggested that PGE_2_ selectively inhibits the differentiation of naïve CD4^+^ T cells into Th1 cells and noted that PGE_2_ can also decrease the production of IL-12 by monocytes or dendritic cells, leading to an effect on the proliferation and differentiation of Th1 cells [37, 38].

### 2.8. PGE_2_ and Th2 Cells

Th2 cells, which mediate humoral immunity, mainly secrete IL-4, IL-5, IL-10, and IL-13. GATA-3 is considered the crucial transcription factor of Th2 cells, autoactivating its expression and driving epigenetic changes in the Th2 cytokine cluster (*IL4*, *IL5*, and *IL13* genes) while suppressing the factors critical for regulating the Th1 pathway, such as signal transducer and activator of transcription factor 4 (STAT4) and the IL-12Rb2 chain [39]. PGE_2_ inhibits the production of both IL-4 and IL-5 by Th2 clones [40]. Later, Bao et al. discovered that PGE_2_ can enhance the IL-4/IFN-*γ* ratio in CD4^+^ T cell culture and polarize CD4^+^ T cells towards Th2 cells [41]. Hence, the promotion effect of PGE_2_ mainly activates the differentiation of Th2 cells via the inhibition of Th1 cells.

### 2.9. PGE_2_ and Th17 Cells

Th17 cell differentiation requires ROR*γ*t, a transcription factor that is induced by TGF-*β* in combination with the proinflammatory cytokines IL-6, IL-21, and IL-23, all of which activate STAT3 phosphorylation. The differentiation of naïve CD4^+^ T cells into Th17 cells is mediated by the EP2 or/and EP4 receptor leading to cAMP production. Moreover, PGE_2_ increases the concentrations of IL-23R and IL-1*β*R. cAMP and other cytokines induce the expression of IL-23R via the EP2 receptor [17, 42]. Klasen et al. found that the expression level of EP4 is significantly increased in Th17 cells from patients with ankylosing spondylitis (AS) compared to those in healthy individuals or rheumatoid arthritis (RA) patients and that the EP4 expression level in Th17 cells from AS patients has a positive correlation with disease activity [43]. Lee et al. declared that the T cell-intrinsic EP2/EP4 signaling is critical in IL-23-driven generation of pathogenic Th17 cells and consequent pathogenesis in psoriasis [44].

### 2.10. PGE_2_ and Tfh Cells

Tfh cells are defined as CD4^+^ T helper cells that express CD40L, chemokine receptor 5 (CXCR5), programmed death 1 (PD-1), and inducible T cell costimulator (ICOS). Bcl-6 is the characteristic transcription factor of Tfh cells [45]. Tfh cells migrate into follicles and interact with antigen-specific B cells to support their differentiation into memory B cells or plasma cells [46]. Tfh cells can secrete IL-21, which binds to IL-21R on the surface of B cells, assists B cell activation and proliferation, and induces self-antibody production. IL-21 is a potent differentiation factor for B cells. Tfh cell defects or hyperactivity can cause immune system dysfunction, leading to the occurrence of autoimmune diseases [47]. A recent study has shown that PGE_2_ can facilitate antibody class switching in B cells by inducing Tfh cell differentiation [48]. We recently discovered that the serum concentration of prostaglandin E metabolite (PGEM) and the proportion of Tfh cells are increased in the collagen-induced arthritis mouse model compared with those in wild-type mice. In addition, there is a positive correlation between the concentration of serum PGEM and the population of Tfh cells in RA patients, suggesting that PGE_2_ acts as a positive regulator of the process of Tfh differentiation [1]. However, the mechanism underlying the regulation of Tfh differentiation by PGE_2_ still needs to be explored.

### 2.11. PGE_2_ and Treg Cells

Tregs are immunosuppressive cells that mainly participate in immune homeostasis by acting as a major barrier to effective immunity against tumors and sterilizing immunity against chronic viral infections [35]. The membrane-bound IL-2 *α*-chain is a marker of Treg cells. There are two critical cytokines, namely, IL-2 and TGF-*β*, that drive the differentiation of Treg cells from naïve T cells. IL-2R is required for both the thymic and peripheral generation of Treg cells. TGF-*β* inhibits the recruitment of Dnmt1, the key maintenance DNA methyltransferase whose activity likely leads to silencing of the newly induced Foxp3 gene [49]. Foxp3 is a specific transcription factor that regulates the development of Treg cells and is essential for the immunosuppressive function of Treg cells, which is initially considered a specific marker of Treg cells. It has been reported that PGE_2_ promotes the growth of Treg cells in both humans and mice via the EP2 or EP4 receptor [50]. Since PGE_2_ can effectively suppress the EP/cAMP/PKA pathway, it also inhibits the differentiation of Treg cells. It is worth mentioning that PGE_2_ signaling via EP2 expressed on human naïve CD4^+^ T cells suppresses Treg differentiation *in vitro* via the cAMP-PKA signaling pathway [3]. However, when UV was used to induce an immunosuppressive reaction, PGE_2_ facilitated an increase in Treg cell numbers. Additionally, Kitipong et al. showed the impairment of the immunosuppressive effect of UV with an EP4 antagonist and the reversal of the indomethacin-induced impairment of immunosuppression by an EP4 agonist. Notably, treatment with an EP4 agonist alone, without UV irradiation, does not result in immunosuppression [50]. Additionally, PGE_2_ inhibits the expression and production of IL-27 by activating conventional dendritic cells *in vivo* and *in vitro*, thus inhibiting the IL-27-induced differentiation and IL-10 production of murine CD4^+^CD49b^+^LAG-3^+^Foxp3^−^Tr1 cells [51]. The imbalance of Th17/Treg may play a role in the progression of autoimmune diseases such as RA [52]; therefore, PGE_2_ is expected to become one of the therapeutic directions.

### 2.12. PGE_2_ and CD8^+^ CTLs

CTLs, which originate from naïve CD8^+^ T cells, can specifically recognize endogenous antigen peptides (i.e., MHC I complex) and induce the apoptosis of target cells. Li et al. showed that CD8^+^ T cell dysfunction correlates with PGE_2_ levels in chronic hepatitis B patients. Patients with high levels of PGE_2_ had more CD8^+^ T cells with remarkably low granzyme B expression and slightly low perforin expression; these proteins are two important effector molecules for the antiviral activity of CD8^+^ T cells in chronic hepatitis B patients [53]. Chen et al. showed that PGE_2_ suppresses exhausted antigen-specific CTL function and promotes CTL apoptosis. The comodulation of PGE_2_ and PD-1 signaling may represent a potent therapeutic avenue for the treatment of chronic viral infections [54]. Kim et al. demonstrated that the elevated mPGES1 expression is associated with low CD8^+^ T cell infiltration into melanomas and poor patient survival [55]. However, concrete results regarding the relationship between PGE_2_ and CTL differentiation are still lacking. Notably, CD4^+^ T cells with cytotoxic activity (CD4^+^ CTLs) have been observed in various immune responses. These cells are characterized by their ability to secrete granzyme B and perforin and to kill target cells in an MHC class II-restricted fashion. CD4^+^ CTLs seem to be derived from various types of CD4^+^ T cells, and several differences have been observed during their differentiation [56]. Most likely, the differentiation of CD4^+^ CTLs can be influenced by PGE_2_.

## 3. Conclusions and Future Perspectives

In this review, we highlight the immunoregulatory roles of the lipid mediator PGE_2_ in controlling the differentiation of T cells. The dysregulation of T cell differentiation leads to the occurrence of various diseases. Although PGE_2_ exerts effects on different CD4^+^ T cell subsets, its role in Tfh cells and CD8^+^ T cells is not well known. The sophisticated interactions between PGE_2_ and T cells still need to be further studied.

Various cells can secrete this lipid mediator, whereas all cells can generate responses to the signaling of PGE_2_ through the EP receptors. Importantly, studying the signaling pathways of PGE_2_ may contribute to further elucidating the pathogenesis of diseases, and targeting PGE_2_ may be used to develop personalized therapies for RA, AS, neuropathic pain, tumors, inflammation, and so on. PGE_2_ may be considered the point of intersection of different human systems. Thus, understanding the pathway of PGE_2_ would yield novel insights for designing more effective therapies for immune diseases.
